# The Philosophy of Chills and Fever

**Published:** 1871-03

**Authors:** 


### THE PHILOSOPHY OF CHILLS AND FEVER.

In a review of Dr. Salisbury's theory of intermittent fever by Dr. John W. Wier, Edwardsville, Ill. (*Boston Med. and Surg. Journal*), the philosophy of chills and fever is thus laid down:

A person exerts himself during a warm day; a cold wind comes on, or night-fall, while he is in a profuse perspiration, and without nourishing food or additional clothing he loses a large supply of animal heat. This is repeated daily for a time; the extremities of the exposed person become cold, he loses vitality, his heart beats feebly, and he sinks into a chill; he is *really dying*, and if there is not vital power enough left, death ensues; if life continues, however, a series of phenomena take place, which, by appropriate treatment, may result in recovery. He does not believe that intermittent fever is produced by any miasmatic poison; but is the result of sudden and repeated draughts of animal heat from the system, which feeble persons can prevent only by suitable clothing, nourishment, and tonics.

